# Feather Pecking and Cannibalism in Non-Beak-Trimmed Laying Hen Flocks—Farmers’ Perspectives

**DOI:** 10.3390/ani9020043

**Published:** 2019-01-30

**Authors:** Eija Kaukonen, Anna Valros

**Affiliations:** Research centre for animal welfare, Department of Production Animal Medicine, University of Helsinki, 00014 Helsinki, Finland; anna.valros@helsinki.fi

**Keywords:** laying hen, egg production, beak trimming, pecking problem, feather pecking, cannibalism, farmers’ attitudes, sustainability

## Abstract

**Simple Summary:**

Pecking-related problems are common in intensive egg production, diminishing hen welfare and production performance, and negatively affecting sustainability. Beak trimming is a common practice to control these problems, but in Finland beak trimming is prohibited. Finnish egg producers have decades-long experience of egg production with intact-beaked hens. This experience, and their management of pecking-related problems, could benefit producers in other countries. The online questionnaire aimed to gather information about Finnish farmers’ attitudes towards beak trimming, their estimation of the seriousness of pecking problems in their laying hen flocks, common risk factors and the best practices to prevent attendant problems. The questionnaire received 35 responses. Finnish egg producers appeared strongly to support a policy of not trimming beaks. Motivation against beak trimming was explained by considering it to be unnecessary and unethical. Most respondents did not regard pecking-related problems as being very severe in their flocks. Lighting, feeding and flock management problems represented the most important risk factors regarding pecking problems. Generally, the same topics were highlighted as being the most important intervention measures for managing an on-going pecking problem. The study indicates that it is possible to incorporate a non-beak-trimming policy as a component of sustainable egg production.

**Abstract:**

Pecking-related problems are common in intensive egg production, compromising hen welfare, causing farmers economic losses and negatively affecting sustainability. These problems are often controlled by beak trimming, which in Finland is prohibited. An online questionnaire aimed to collect information from farmers about pecking-related problems in Finnish laying hen flocks, important risk factors and the best experiences to prevent the problems. Additionally, the farmers’ attitudes towards beak trimming were examined. We received 35 responses, which represents about 13% of all Finnish laying hen farms with ≥300 laying hens. The majority of respondents stated that a maximum of 5–7% incidence of feather pecking or 1–2% incidence of cannibalism would be tolerable. The majority of respondents (74%) expressed that they would definitely not use beak-trimmed hens. Only two respondents indicated that they would probably use beak-trimmed hens were the practice permitted. Among risk factors, light intensity earned the highest mean (6.3), on a scale from 1 (not important) to 7 (extremely important). Other important problems included those that occurred during rearing, feeding, flock management and problems with drinking water equipment (mean 5.9, each). The most important intervention measures included optimal lighting and feeding, flock management, and removing the pecker and victim. Concluding, Finnish farmers had strong negative attitudes towards beak trimming. The study underlines the importance of flock management, especially lighting and feeding, in preventing pecking problems and indicates that it is possible to incorporate a non-beak-trimming policy into sustainable egg production.

## 1. Introduction

Feather pecking and cannibalism are common problems in intensive egg production, compromising hen welfare [1,2] and causing economic losses to the farmers [3,4,5]. Pecking at flock-mates can roughly be divided into gentle feather pecking, severe feather pecking and cannibalism [6]. According to research, gentle feather pecking is most common at a younger age, while severe feather pecking [7,8] and cannibalism occur more among older birds [8].

There are multiple factors causing feather pecking and cannibalism. Different breeds have differing propensities towards pecking [9,10]. It is possible to reduce pecking problems through genetic selection [11,12], which have also been connected with fearfulness of birds [9,13,14]. Feeding related issues may be linked to pecking problems and changing diets more often increases the risk for feather pecking [15,16]. Providing pelleted feed appears to increase the risk for feather pecking, whereas mash feeding reduces the risk [17,18]. Higher fibre content in the diet decreases cannibalism [19].

The likelihood for pecking problems varies among different production systems. Pecking-related problems are more common in aviary systems, deep litter systems and enriched cages compared with traditional cages [1,2]. On the other hand, aviary systems seem to be less risky compared with a floor system, probably because multiple tiers in aviary systems offer greater possibilities for pecked birds to escape the peckers [14]. In addition to production system, management competence is also involved [2]: litter condition [8,14], light intensity [9,14], stocking density [20], temperature and humidity [16] greatly influence pecking behaviour.

Management during the rearing period strongly affects pecking behaviour [16]. Apparently, severe feather pecking during the rearing period increases the risk for pecking problems in adult hens [14]. Hence, pecking-related problems during the production period may reflect feather-pecking patterns adopted during the rearing period [16].

Offering different types of enrichment may reduce the occurrence of pecking-related behaviour [21,22,23,24]. Earlier studies suggested that feather pecking is misdirected foraging behaviour [25] and, therefore, promoting foraging behaviour has been linked with reduced feather pecking [18,22,26]. Offering litter material already at a young age seems to decrease the incidence of pecking problems later in life [23]. Furthermore, enriching the rearing environment with litter material reduces fearfulness at an older age [27] and, thus, may reduce the incidence of pecking problems. Moreover, the possibility to perch early in life appears to lower the risk for vent pecking during the laying period [28].

A common means to control pecking-related problems has been beak trimming, in other words, cutting off the tip of beak [1]. The procedure appears to be efficient, because pecking-related problems are more common, and feather condition is worse among birds with intact beaks compared with beak-trimmed birds [7,19,29,30]. Nonetheless, these misbehaviours also exist in beak-trimmed flocks [7,8,15,24] and, thus, beak trimming fails to completely prevent pecking-related problems. Furthermore, beak trimming has negative consequences on the natural behaviour of the bird: beak-trimmed hens exhibit less ground pecking [29] and preening [31], and the procedure is painful [32]. Neuroma formation on the tip of the trimmed beak can also lead to long-term pain [32]. The cause of pecking problems is not the beak, as such: feather pecking and cannibalism arise due to various stressors [1,9]. Beak trimming, therefore, does not solve the underlying problem but only addresses the consequence. Therefore, raising intact-beaked hens is an integral part of sustainable egg production.

In 1999, the European Union prohibited all mutilations of laying hens, except beak trimming before the age of 10 days in order to prevent pecking-related problems [33]. Therefore, beak trimming has, until recently, been a routine procedure in egg production. However, some European countries, such as the UK, Germany, and the Netherlands, have prohibited or aim to prohibit beak mutilation, while some countries, such as Finland and Sweden, already have implemented a full ban.

Beak trimming was prohibited by law in Finland in 1996, but even before the ban it was never a routine procedure in Finnish egg production. According to poultry experts in Finland (personal communications), only brown laying hens were occasionally beak trimmed before the ban. Hence, Finnish egg producers have decades-long experience of egg production with hens with intact beaks. This experience and the attitudes of Finnish egg producers towards beak-trimming or non-beak-trimming policy, and their means for managing pecking-related problems, could benefit producers in other countries but have not to date been collated.

The study aimed to gather information about the level of feather pecking and cannibalism in Finnish commercial laying hen flocks as estimated by farmers, the most common risk factors for pecking problems, and the best practices to prevent these problems. In addition, the farmers’ attitudes towards beak-trimming were examined.

## 2. Materials and Methods

An online questionnaire was distributed to Finnish laying hen farmers during winter 2017–2018. The questionnaire was advertised in the Finnish poultry journal before it was made available. The Finnish Poultry Association distributed the questionnaire via egg packaging centres and social media channels to Finnish egg producers. In Finland, there are 270 laying hen farms with ≥300 laying hens. The association estimated that the questionnaire reached about 90% of Finnish egg producers. It was open for a month.

The study defined feather pecking as pecking that damaged feather cover, leading to broken feathers and feather loss (slight skin damage caused by feather plucking may occur). Cannibalism was defined as pecking that damaged skin and underlying tissues.

The questionnaire comprised 43 questions, most of which were asked on a farm basis, but if the answer was expected to differ among flocks, the answers were collected on a flock basis (Table S1). First, we gathered general information about the farms, the production system and the birds. The next topic included questions on farmer attitudes towards beak trimming, and farmers’ estimation about the seriousness of existing pecking problems, as well as the effect of pecking-related problems on production performance. The seriousness of pecking-related problems was assessed on a scale from 1 (not serious) to 7 (extremely serious). The attitude towards beak trimming was assessed using a scale from 1 ([I would] most definitely not) to 7 ([I would] most probably) use beak-trimmed hens if beak trimming were legal. Many of the questions focused on the farmers’ opinions of the importance of a range of risk factors. These were selected based on the literature and practical experience, as being known to influence pecking problems, and included breed, production system, the origin of the birds and rearing period, housing conditions and bird management, perches, nests, litter, feeding, unexpected problems, and health-related issues. In addition, we clarified opinions on optimal age of pullets for transportation, detailed information about suitable lamps and litter, problems with perches and nests, and preferable time spent on observing birds with open-answer questions. The last topic investigated the farmers’ estimates, based on practical experience, of the most effective preventive measures (additional enrichment, such as straw bales, hanging objects and pecking stones) and means available to manage on-going pecking problems (i.e., intervention, such as removal of the pecker or victim from the flock). All the estimations used a scale from 1 (not important) to 7 (extremely important). Additionally, at the end of the questionnaire, the respondents were asked to, in their own words, and in order of importance, list the five most important measures, to prevent pecking problems and manage on-going problems (intervention measures). To rate all mentioned factors, the most important factor received five points, and the least important factor one point. For analyses, all points were summed to obtain total points given to each factor. Finally, the respondents were able to present free opinions concerning all topics.

### Statistical Analysis

All statistical analyses were carried out with SPSS vs 24 (Armonk, NY, USA). Since most data did not meet the assumptions of normality, and most variables were not continuous, all data were analysed with nonparametric tests. Since the median in such data is less informative and does not differentiate the measured risk factors sufficiently, we provide the descriptive results both as means with standard errors, and medians with minima and maxima. Differences between conventional and organic production were analysed with the independent samples Mann-Whitney U-tests. Differences between production systems (small enriched cages accommodating <20 hens, large enriched cages accommodating >20 hens, floor system, aviary system, ranging possibility) were analysed using the independent samples Kruskal-Wallis test, and further adjusted pairwise significance levels with Bonferroni-correction. Only statistically significant differences (*p* < 0.05) are reported.

## 3. Results and Discussion

We received a total of 35 responses, of which seven (20%) were from organic farms. Most farms (20, 60%) had a single hen house. The number of houses (and, thus, flocks) per farm varied between one and five. Altogether, we received information on 58 flocks, of which 14 were organic flocks. The number of farms represents about 13% of 270 Finnish laying hen farms with ≥300 laying hens, and 15% of the farms that were estimated to have been reached by the questionnaire. In organic farms, the average flock size was 2700 ± 150 hens (median 3000 hens, 1100–3000 hens), and in conventional production 17400 ± 1600 hens (median 16,100 hens, 1400–49,000 hens). The most common flock size was 3000 hens (nine houses, 16%). The majority of respondents (31, 88%) represented solely egg producing farms, and the remaining four farms had pullet rearing in addition to egg production. According to current statistics [34], in Finland, nearly half of laying hen flocks (47%) consist of 10,000– < 30,000 birds, and 38% of the flocks are larger. The maximum legal size of an organic laying hen flock is 3000 birds [35]. We thus assume that the respondent farms represent Finnish egg producing farms reasonably well. Due to the low response rate, the results should be considered with some reservation. The low response rate may reflect attitudes of Finnish farmers towards pecking problems: Pecking-related problems are not considered very important. Further, the results may be influenced by voluntary participation, which always affects the selection of responses.

The respondents had a long experience of egg production, on average 18 ± 1.7 years, ranging from 1.5 to 40 years. The production equipment was installed in hen houses between 1995 and 2016, most commonly in 2010 (14 houses, 24%). On most farms, hens were kept in enriched cages (26 farms, 45%), of which 22 (85%) had large enriched cages for groups of >20 hens and the remainder had small enriched cages with groups of <20 hens. An aviary system was used in 18 (31%) of the houses and a floor system in 14 (24%) of the houses. In organic production, eight flocks were in floor houses and six in aviary systems. One third of the respondents (11, 32%) stated having experience of different production systems. Unfortunately, we did not ask of which different systems with which the respondents had experience. To a certain extent, we can assume that most respondents had experience of traditional cage systems because, before 2012, nearly 80% of eggs were produced in traditional cages [34]. Altogether 14 respondents estimated the differences between production systems with respect to pecking problems. In these responses, enriched cages were most often (12 responses) considered to be the easiest. There were different opinions on which system is the most difficult system, with respect to feather and cannibalistic pecking, and all systems were mentioned at least once. However, floor systems were most commonly, in seven responses, considered to be the most challenging system.

In Finland, in 2017, 62% of table eggs sold via egg packaging centres (74% of total egg consumption) were produced in enriched cages [34]. The farms with enriched cages are generally larger compared with the other production systems and, therefore, the proportion of farms with enriched cages is lower (40%) than the total number of laying hen farms. Aviary systems are used in 21% of the Finnish laying hen farms and floor systems in 31% of farms [36]. Together, deep litter and aviary systems account for about one third (32%) of the total egg production [34]. However, the statistics show a trend of decreasing production in enriched cages and increasing production in aviary systems [36]. Currently, organic production covers only about 5% of all egg production [34] and represent 15% of egg producing farms because these farms are generally smaller compared with the other production systems [36]. To date, free-range production is practiced in only 8% of egg-producing farms [36]. In the questionnaire responses, the proportion of organic production appears to be slightly overrepresented. Nonetheless, we can assume that the respondent farms represent the Finnish situation reasonably well.

Due to climatic conditions, outside ranging poultry represents a minority in Finnish egg production. Possibly, due to minimal experience, the respondents did not indicate that free-range or organic systems represented particularly difficult systems. According to earlier research, severe feather pecking has been reported to occur more commonly and severely among organic poultry [8]. However, systems with outdoor range are also considered as to reduce the risk for pecking, and pecking-related problems decrease along with increased outside range usage [7,15,24,37]. On the other hand, in many countries, the situation in relation to pecking problems may differ considerably between organic and conventional production because beak trimming is not as common a practice in organic egg production as it is in conventional production [8]. Severe feather pecking appears more regularly among non-beak-trimmed birds [1,7] resulting in worse plumage condition [7,29,30]. In Finland, all egg production is with hens with intact beaks, consequently equalizing the production systems from that perspective.

The banning of beak trimming in Finland apparently had no major effect on laying hen management because all respondents stated that they had not changed their management due to the ban on beak trimming. This is understandable because even when beak trimming was allowed it was never adopted as a routine procedure in Finnish egg production and thus the change in the law was fundamentally without effect on the lives of poultry farmers. Although the respondents had long experience of egg production, not many had been producing eggs before the ban. Yet, on many farms, egg production may have been practised before the current farmer, and the experience of the former farmer could have been transferred to the current farmer. This assumption is supported by a comment of a farmer, with 18 years of experience of conventional egg production:

“There have been no disadvantages of the non-beak-trimming policy. On our farm, we have had chickens for over 40 years without pecking problems.”

The vast majority of respondents (26, 74%) declared that they most definitely would not use beak-trimmed hens even if it were allowed (Table 1). Motivation against beak trimming was explained by considering it to be an unnecessary and unethical procedure. A couple of respondents also stated that using non-beak-trimmed hens boosted the image of egg production, and that beak trimming would cause unnecessary costs. A farmer, with 29 years of experience in conventional egg production, stated that:

“I do not know why we should torment the animal when there are no advantages gained by beak trimming. Only expenses and suffering.”

Only two respondents indicated that they probably would use beak-trimmed hens were it permitted. One of these respondents did not provide any explanation for the opinion, while the other supported beak trimming because it would make management easier.

The Finnish egg producers’ attitudes appear to strongly support non-beak-trimming policy, but not without discord. As a comparison, tail docking in pig production is also banned and the majority of Finnish pig farmers (61%) support non-tail-docking policy [38]. The consensus among egg producers seems even stronger than among pig farmers. Finnish pig farmers motivated their stance against tail docking with ethical arguments [38], thus using similar reasons as the egg producers in the current questionnaire. Among Finnish pig producers, tail docking was often supported for economic reasons and because it made management easier [38].

### 3.1. Seriousness of Pecking Problems

On the scale from 1 (not serious) to 7 (extremely serious), the seriousness of feather pecking on respondent farms was estimated on average as 2.2 ± 0.22 (median 2, 1–6), and cannibalism 1.7 ± 0.18 (median 1, 1–5) (Table 2). In 9 (26%) of the responses, 5–7% incidence of feather pecking was considered tolerable, while 23 (66%) of the responses stated that, at its highest level, an incidence of 5–7% would be tolerable. Attitudes towards cannibalism were more critical. Nearly half of the respondents (17, 49%) expressed that 1–2% incidence of cannibalism would be tolerable, and 10 (29%) of the respondents stated that cannibalism, at any level, would not be acceptable. No one was willing to accept > 8% incidence of cannibalism.

Finnish egg producers consider cannibalism among hens to be a less serious problem than do pig farmers with respect to tail biting, according to the above-mentioned study (86% of the responding egg producers and 72% of the pig producers [38] replied with scores 1 and 2). However, the same proportion of the respondents (29%), in both studies, indicated that no cannibalism would be acceptable.

Half of the farmers (16, 50%) stated that pecking problems typically occur during peak production. According to the experience of eight (25%) of the respondents, the typical time for pecking problems is at the beginning or at the end of the production period.

Several studies show an increase in severe feather pecking with increasing age [5,7,8,14]. According to a questionnaire survey among egg producers in the UK, feather pecking most commonly starts at around 40 weeks of age [15]. The experience of half of the respondents in the current survey disagrees with those results because peak production was identified as being the riskiest period. It might be that at around peak production the problems typically start, and the starting point requires more effort to get the situation under control. Even if the problem continues thereafter, the farmers may not consider it to be such a severe problem any longer. The stress caused by transport from the rearing farm to the laying farm can initiate cannibalistic pecks, which might be one reason for the perceived risky stage being early in production [39]. However, in the current study, the beginning of production period was not named as the riskiest time for pecking problems.

Most respondents (31, 89%) estimated that at highest 5–7% of the hens in their current flock(s) showed signs of feather pecking. The majority of respondents estimated that the occurrence of feather pecking in the current flock(s) was at a usual level (33 flocks, 63%), while in four flocks (8%) the occurrence to exceed the usual level, and in 15 flocks (29%) feather pecking occurred less commonly than usual. Furthermore, 43% of the respondents estimated that feather pecking had not occurred in any flocks during the last five years at a level higher than what was perceived tolerable. Only two farmers (6%) estimated that >50% of the flocks during the last five years had shown signs of feather pecking at higher than tolerable levels. Organic farmers estimated that feather pecking occurred more often in their flocks during the last five years at a higher than tolerable level compared with conventional production (median in organic farms 30% (range 0–65%) and in conventional farms 0.01% (range 0–20%); *p* = 0.006, independent samples Mann-Whitney U-test).

Regarding cannibalism, the majority of respondents reported that cannibalism did not occur in their current flock(s) (14, 77%), while in nine current flocks (17%) 1–2% of the hens showed signs of cannibalism. According to farmers’ estimations, signs of cannibalism are more common in organic than in conventional production (median in organic farms 0.5%, range 0–7%, and in conventional production 0%, range 0–6%; *p* = 0.004, Mann-Whitney U-test). In the current flock(s), the incidence of cannibalism was estimated to be at a usual level for 26 (50%) of the responses, higher than usual for six (12%) and less than usual for 20 (38%) of the responses. According to 21 (60%) of the responses, cannibalism had not occurred in any flocks during the last five years more frequently than was estimated to be tolerable. However, two responses estimated that in 30% of the flocks during the last five years cannibalism had been a more common problem than was considered tolerable.

Overall, the questionnaire responses reveal that the level of existing pecking problems in Finnish laying hen flocks, in farmers’ opinions, seems fairly low. However, had the investigation been made by researchers the findings might have differed. Despite the given definitions for feather pecking and cannibalism, the estimation of individual respondents probably varied considerably. Indeed, in free comments, one respondent admitted having difficulties identifying the connection between feather cover condition and pecking problems. Farmers may consider poor feather cover in older hens as a normal course of a hen’s life without realizing that it might be due to feather pecking. Surely, feather pecking is not the only factor affecting feather condition towards the end of production period, but it most probably is partly responsible for the situation. Also, small skin lesions caused by cannibalistic pecking could have remained undetected by farmers because this would have required picking up hens for thorough investigation. We did not ask or advise farmers to perform such a detailed examination and, most probably, the estimation was thus based on an approximate scanning during routine flock checking.

Not many studies have canvassed farmers’ points of view. A study conducted in the UK reported that 65% of the flocks showed feather pecking according to farmers’ estimations. In the same study, the researchers detected severe feather pecking in 73–86% of the flocks [7]. An earlier study reporting farmers’ estimation in the UK informed that 57% of the flocks showed feather pecking behaviour and 47% of the farmers described feather pecking to be a normal phenomenon in their laying hen flocks [15]. In studies performed in commercial conditions, severe feather pecking is typically seen by researchers in 55–86% of the hens [7,8] and cannibalism in 21–37% [8]. A Dutch study reported that in nearly half of the examined flocks over 10% of the hens showed severe feather damage [14].

### 3.2. Production Losses

In most responses (19, 54%), pecking problems were not typically considered to cause losses in egg production, while four (11%) estimated losses to reach, at its highest level, 11–20%. Most commonly, in 11 (31%) of the responses, losses were estimated not to exceed 1–5%. Half of the responses (51%) assessed mortality due to pecking-related problems to average 1–5%. Most commonly the respondents (15, 43%) estimated that mortality due to pecking problems did not exceed 1–5%, while 12 (34%) claimed that pecking problems did not result in any mortality. One farmer, however, estimated that mortality could be as high as 11–20%.

The farmers’ estimations about losses of egg production and mortality due to pecking problems appear to be moderate and, thus, are in line with their estimations of the level of existing pecking problems. Feather pecking does not typically lead to mortality or culling [5,15], but severe cases of cannibalism increase mortality [4,19,40]. According to reports, feather pecking [3,5] and cannibalism [4] negatively affect egg production, and feather loss due to pecking-related problems increases feeding costs [3], consequently leading to reduced income, thus negatively impacting sustainability.

### 3.3. Risk Factors

All lighting-related issues included in the questionnaire were considered extremely important regarding pecking problems (Table 3). The risk factor that was rated highest among all risk factors was light intensity, with a mean of 6.3, and no one estimated the effect to be less than 4. Free comments emphasized that when choosing the light source (type of lamps) one should take into account, in addition to intensity, light colour, flickering and the possibility to use dimming.

The importance of light intensity on pecking behaviours has frequently been reported: bright light is associated with increased incidence of pecking problems [9,14,41], while dim light reduces the incidence.

Laying hen farmers often blame direct and indirect sunlight to provoke pecking problems. In organic farming, natural light is unavoidable both inside and outside. Surprisingly, attitudes towards lighting did not differ between organic and conventional farms, not even towards natural light. This could suggest that, in both systems, natural light is considered to be an equal contributing factor. However, an organic farmer noted that:

“Dusky areas are needed, while even lighting in feeding and foraging area is good. However, during times of outdoor access, external bright natural light does not seem to cause problems.”

This could mean that farmers consider that natural light, via windows, in organic laying hen houses creates disturbing bright spots that could trigger pecking problems, whereas sunlight in outdoor areas, creating shadow and light variation, is welcomed, causing no harmful effects. The effect of natural light on pecking behaviour of laying hens, however, requires further research.

Overall, topics related to feed and drinking water were considered utmost important in the responses, all with means ≥ 4.5.

Feeding-related issues are typically acknowledged in studies exploring risk factors affecting feather pecking. Feeding and drinking equipment and inadequate access to feeders increases feather pecking [15]. Changing diets more often over the rearing period [16], or during laying [15], increases the likelihood of feather pecking during the production period. Among Finnish farmers, however, changing diets got a moderately low mean score (4.9) compared with other feeding-related issues. We cannot make further conclusions about that matter because we did not ask how many times the diet was changed on the responding farms. Feed form and composition also impact feather-pecking behaviour in laying hens. Mash feeding reduces feather pecking, probably because it increases time spent eating and encourages foraging behaviour better than when feed is in a pelleted form [17,18]. Diets with higher fibre content are associated with lower mortality resulting from cannibalism [19].

The importance of farmers’ and/or caretakers’ management competence earned a mean score of 5.9 and was highlighted throughout the responses. Apparently, it is difficult to estimate time spent on observing birds because we received only 11 responses. According to these estimates, time spent on observing birds ranged from 25 minutes to two hours per house, being one hour in four responses, and two hours in three responses. Surprisingly, flock size was not related to time spent observing. The results, thus, indicate that a larger flock may be checked faster under one farmer’s responsibility than a smaller flock under another’s.

The influence of production system on the likelihood of pecking problems was estimated to be important, with a mean of 4.9. Risk of pecking problems varies according to production system. In enriched cages, pecking-related behaviour is less frequent than in floor and aviary systems [2]. An aviary system seems to be less risky compared with a floor system, probably because the multiple tiers in aviary systems offer more possibilities for pecked birds to escape the peckers [14]. In a free-range system, feather pecking is less frequent, and plumage condition appears better, whereas cannibalism, in form of vent pecking, occurs more commonly [42]. Larger group size increases the likelihood of feather pecking [43] that may partly explain the differences between the production systems.

According to responses, air quality and temperature represent important risk factors, with means over 4 (Table 3). Extremely hot summer weather was important according to the responses (mean 5.1), power failures likewise (mean 4.9). Thunder weather, however, divided opinions between conventional and organic production farmers. Organic farmers seemed to worry less about thunder (median 2, range 1–5) than conventional farmers (median 4, range 1–7; *p* = 0.039, Mann-Whitney U-test).

The practical experience of Finnish laying hen farmers supports earlier reports that repeatedly identify the maintenance of housing conditions as being an important factor influencing pecking problems. Managing housing conditions, such as litter [8,14,15], ventilation, temperature and humidity [15,16] also influences pecking behaviour. A house temperature of less than 20 °C is associated with a higher risk of feather pecking [15]. In Finland, modern hen houses are typically equipped with heating and ventilation systems [44] that better ensure uniform temperature. Under summer heat, maintaining house temperatures at optimal levels is challenging. This explains why hot summer weather was associated with a high mean. Season, in contrast, was considered to be a minor risk factor in the current survey.

Furthermore, our results indicate that hen density plays an important role for pecking problems (mean 5.3). We established differences in attitudes towards bird density between production systems. Density was considered more important in farms with large enriched cages compared with farms with small enriched cages (median for large cages 6, range 3–7, and for small cages 3, range 2-4; *p* = 0.037, Kruskal-Wallis test). In addition, farms with small enriched cages and a floor system differed (median for small cages 3, range 2–4, and for floor system 7, range 4–7; *p* = 0.008, Kruskal-Wallis test). Some studies report that pecking problems occur more often at higher bird densities [20], while in other studies the link between stocking density varies with age and group size [45].

In the open answers, some farmers pointed out that stress of any type is an important risk factor. Undoubtedly, stress is an essential risk factor increasing a range of health and welfare problems, including pecking [18]. However, without specifying the stressor, it remains too nonspecific to offer practical assistance in solving the problem. Yet, by mentioning stress as a risk factor the respondents may simply have highlighted the range of possible factors predisposing to pecking problems [1,2,9], and communicated an important message that everything must be taken into account if pecking-related problems are to be prevented.

Bird health-related issues generated high means in our questionnaire, all associated risk factors yielding a mean of ≥5.2 (Table 3). An earlier survey showed an increased risk for pecking problems when the flock suffered from infectious bronchitis (IB, a Corona virus infection) and egg peritonitis (generalized bacterial peritonitis during laying period, typically caused by *Escherichia coli*). The same survey also established a connection between parasites and pecking problems [15] that was also considered an important risk factor in the current study (mean for ectoparasites 5.4 and for endoparasites 5.2). Immune responses induced by vaccinations or infections are related to increased feather pecking [46]. In most countries, laying hens are heavily vaccinated. However, the poultry disease situation in Finland remains relatively good [47] and, therefore, also the vaccination programme is modest compared with those in most European countries. For example, in Finland, laying hens are not vaccinated against IB or Newcastle disease. We could argue that the good health situation, and consequent minimal vaccination programme, lowers the risk for pecking problems in Finnish circumstances.

According to the responses, success in pullet rearing impacts the occurrence of pecking problems. Problems during the rearing period had a high mean (5.9). Additionally, the poor uniformity of body weights of the pullet flock and the source of the birds were considered important (means 5.6 and 5.1, respectively). Signs of feather or skin damage in transported pullets were, however, only seldom (20, 57% of the responses) or never (15, 43% responses) observed. This is understandable because during the rearing period a hen moults several times, changing its feathers [48]. Furthermore, as discussed previously, small skin damages are difficult to detect without thoroughly examining individual birds.

Our questionnaire did not focus on rearing period and we therefore cannot reach more detailed conclusions about the risk factors involved in the rearing period. However, the practical experience of Finnish egg producers supports several previous studies outlining the importance of rearing period on pecking problems during the laying period. Chicks at a young age can express all manner of pecking behaviour, including severe feather pecking [5,14,16] and cannibalism [4]. If the birds have adopted a feather-pecking habit during rearing, the flock is at a higher risk of feather pecking during the laying period [4,5,14,16,41], making it important to prevent pecking problems already at a young age. Enriching the rearing environment with litter material, to allow foraging, reduces pecking problems during the laying period [4,14,16,23,28]. Furthermore, offering a perching opportunity at a young age, before four weeks, reduces the risk of vent pecking [14,28].

The feedback from the current questionnaire indicates that Finnish egg producers do not think that litter material represents a substantial risk factor in relation to pecking problems (mean 3.4). Litter condition, however, was deemed more important (mean 4.1), probably reflecting the respondents’ practical approach to the matter. In practice, litter condition, plays a more important role than litter material as such.

Earlier studies suggest that feather pecking is misdirected foraging behaviour [25,49]. Bedding material and condition affect the litter-related behaviour of poultry. Friable litter allows birds to express natural litter-directed behaviour more easily, including foraging, scratching and dustbathing [26,50]. Foraging and scratching bedding depend on age and available material; young chicks prefer wood shavings while, over time, birds start to express more interest towards sand [51]. Litter is of little value in enriched cages, compared with floor systems, and that most likely explains the low mean in this study. However, also in enriched cages, foraging possibility improves plumage condition [52]. Green et al. (2000) [15] observed a higher risk for feather pecking when friable litter was absent at the end of the production period. The study of de Jong et al. (2013) [51] found no differences among different bedding materials (sand, paper and wood shavings) in relation to feather pecking.

On average, the importance of location and functionality of perches and nests were reported to be below 4, thus representing negligible risk factors. Yet, it appears that some farmers have thought seriously about the perching behaviour of chickens. Some respondents emphasized in their open answers, the importance of perches by pointing out that there should be enough, they should be correctly located and well-designed such that hens willingly use them. A couple of respondents commented that the high usage of perches lowers the bird density pressure elsewhere. An organic farmer, with 20 years of experience, commented that:

“There have to be enough perches (over the legal requirement), the kind of perches that hens actually use (not in drafty places etc.) and can reach for a daytime snooze and escape onto.”

In the free comments, the importance of nests was explained by a couple of respondents. Sufficient numbers of nests are required so that hens do not need to queue for the nests. Additionally, one should pay attention to the size, safety and accessibility of the nests because mistakes with these issues are apt to cause problems.

Offering a possibility to perch already early during the rearing period decreases pecking problems later in life [14,16,28]. Enabling perching on high perches reduced pecking at flock mates and feather damage in a Swiss study [53]. The relevance of perches and nests is probably considered lower in enriched cages, which may explain why perches and nests were associated with low means in the current questionnaire. The nest design may affect feather pecking. A questionnaire among egg producers in the UK suggested that offering communal nests for hens increases the likelihood of feather pecking [15]. Another study found that darkness in the nests reduces the incidence of feather pecking [44].

Breed as a risk factor behind pecking problems was estimated to exceed 4 (Table 3). According to the responses, 98% of respondents had white breeds (37% Lohmann LSL-Classic, 26% Lohmann LSL-Lite, 21% DeKalb and 14% H&N Nick Chick) and only one flock was brown (Brown Nick). About 90% of laying hens in Finland are white [45], thus, our responding farms correspond well with the general situation. Over half (54%) of the respondents had experience of different breeds, and 75% said that they always choose the same breed. In free comments, continuously choosing the same breed was often rationalized with the permanent relationship with the rearing farm and with good experience of the breed over the years. A common reason to change the breed was often to search for suitable egg size. The behaviour of the breed and pecking-related problems were not commonly mentioned in responses. However, “good experience” probably includes these issues.

### 3.4. Preventive Measures

According to the lists of respondents, the most important measures to prevent pecking problems were optimal light and feeding (Table 4). Both received 12 first places, but in total rating, feeding earned higher points. Two respondents listed housing conditions as being most important. Additionally, avoiding natural light leakage into the hen house was twice considered to be the most important factor preventing pecking problems. However, in terms of total points, housing conditions rated considerably more important than natural light. Flock management was deemed most important once.

In free comments, some farmers emphasized the importance of good housing conditions in preventing pecking problems rather than beak-trimming policy. For example, a farmer with 15 years of experience in conventional production commented:

“There is no need [to beak trim] because hens do not peck one another when the housing conditions are good and there are no stressors.”

An earlier questionnaire among farmers in the UK [15] revealed that similar preventive measures are in use in both countries. Managing housing conditions was also emphasized in the UK survey. However, although beak trimming was mentioned as one option [15], beak trimming does not completely prevent pecking-related problems [7,8,15,24]. Furthermore, feather pecking and cannibalism can arise in the presence of various stressors, including suboptimal housing conditions [1,9], and, thus, may be, in a sense, a hen’s way of indicating inadequacies. Therefore, beak trimming does not solve the underlying problem but only addresses the consequence.

Overall, among the respondents, additional enrichment was of low value in preventing pecking problems (Table 5). Only for straw bales was a mean of > 4 reached. One reason could be that over half of the farmers had no experience of such enrichment, probably because most hens were kept in enriched cages. Straw bales were not used in 63% farms, 74% had no experience of pecking stones, and 63% had not used hanging strings, and 54% other types of hanging object, as enrichment. Straw bales were more important among floor houses as compared with houses equipped with enriched cages accommodating >20 hens (median for floor houses 3.5, range 0–7, and for enriched cages 0, range 0–3; *p* = 0.002, Kruskal-Wallis test). This finding is understandable because it would be impossible, or at least very impractical, to use straw bales in cages. Additionally, the importance of hanging objects differed between housing types (*p* = 0.032), but statistically significant differences were absent in a pairwise comparison.

Offering birds enrichment, such as novel objects or bedding material, seems to reduce feather pecking [22,23]. Enriching the birds’ environment with hay bales reduces gentle feather pecking more than a plastic box, but hay bales did not reduce severe feather pecking [54]. A survey observing commercial farms over two consecutive flocks reported less severe feather pecking in the latter flock due to increased enrichment [24]. However, in addition to enrichment, the close follow-up of the farms by the researchers, in the earlier study, could have improved the management of housing conditions and, thus, led to decreased pecking problems. The low valuation of enrichment among Finnish egg producers in the current survey does not necessarily mean that they do not appreciate offering a furnished environment to birds but that, according to their experience, the foundation must be properly arranged in the first case, and enrichment should represent a secondary measure only.

### 3.5. Intervention Measures

Removing the pecker from the flock was perceived as more important than removing the victim (Table 5). However, removal of the victim was more important for conventional farmers than for organic farmers (median for conventional production 4.5, range 1–7, and for organic 3, range 1–5; *p* = 0.025, Mann-Whitney U-test).

According to the open-worded measures listed by respondents, the most important intervention to manage a flock with on-going pecking problem was to ensure the correct light conditions (Table 4), and immediately accomplish necessary adjustments, such as to reduce light intensity, change light colour and replace broken lamps. The second important issue was to check and, if necessary, optimize feeding. Maintaining optimal lighting and feeding were priorities in both listings of farmers, along with preventive measures and a to-do-check list with an on-going pecking problem.

Two responses mentioned careful flock observation and instant reaction to make essential corrections, if any signs of unwanted behaviour become evident. Additionally, removing the pecker and victim earned first places twice, the removal of the pecker securing slightly higher total points. Presenting feed or other foraging material, adjusting housing conditions, including ventilation, and offering additional salt (NaCl) were given first place once each.

From an individual bird perspective, it is important to separate the victim to either nurse or cull the injured bird, but from the flock point of view, removing the pecker may be a more efficient way to respond. One pecking bird can damage many birds, and pecking behaviour appears to spread by mimicry [55]. Therefore, the removal of the pecker reduces this risk.

Damaged feathers attract to peck each other’s feathers and a hen with damaged feathers is prone to become a victim of cannibalism [40]. If the first signs of pecking problem are missed without appropriate action, the battle is easily lost and pecking may spread rapidly throughout the whole flock leading to high mortality through cannibalism [40]. That is to say, the fact that there is little, if any, room for error further emphasizes the importance of a farmer’s ability to offer and sustain an adequate environment for hens. Cannibalism can break out because of several predisposing factors acting together [40] and, therefore, intervention measures often must be combined. A survey conducted in the UK listed the farmer intervention measures in cases where pecking problems had started [15]. Several factors mentioned in their list were similar to those Finnish farmers referred to. Dimming lights was the most common factor in both questionnaires, as was the use of red lights, removing peckers and providing additional salt. The measures that were used in the UK, but which were not mentioned by Finnish farmers were water spray over the birds and provision of additional vitamins. Additionally, beak trimming was included in the tool box of the UK farmers [15].

## 4. Conclusions

In this questionnaire study, the Finnish laying hen farmers strongly presented negative attitude towards beak trimming and the respondents did not generally regard pecking-related problems as being very severe problems in their flocks. The study also reveals that the main focus should be on managing housing conditions, especially lighting, and ensuring optimal feeding when preventing or handling an existing pecking-related problem. In conclusion, the study indicates that it is possible to incorporate a non-beak-trimming policy as part of sustainable egg production.

## Figures and Tables

**Table 1:** The Finnish laying hen farmers’ (*n* = 35) attitude towards beak trimming, on a scale from 1 ([I would] most definitely not) to 7 ([I would] most probably) use beak-trimmed hens if beak trimming were legal.

Scale	Frequency	Percent (%)	Cumulative Percent (%)
1	26	74.3	74.3
2	4	11.4	85.7
3	1	2.9	88.6
4	2	5.7	94.3
5	0	0	94.3
6	0	0	94.3
7	2	5.7	100

**Table 2:** The seriousness of feather pecking (a) and cannibalism (b) estimated by Finnish laying hen farmers (*n* = 35), on a scale from 1 (not serious) to 7 (extremely serious).

Scale	Feather Pecking		Cannibalism	
	Frequency	Percentage (%)	Frequency	Percentage (%)
1	12	34.3	20	57.1
2	13	37.1	10	28.6
3	4	11.4	1	2.9
4	4	11.4	3	8.6
5	1	2.9	1	2.9
6	1	2.9	0	0
7	0	0	0	0

**Table 3:** The importance of risk factors for pecking-related problems in laying hens, detailed by Finnish egg producers.

Risk Factor	Mean *	Median (Range) *
Light intensity	6.3 ± 0.16	7 (4–7)
Problems during rearing period	5.9 ± 0.21	6 (4–7)
Mistakes in feed composition	5.9 ± 0.22	6 (3–7)
Farmer’s management skills	5.9 ± 0.23	6 (3–7)
Problems with drinking equipment	5.9 ± 0.25	6 (2–7)
Problems with feeding equipment	5.8 ± 0.25	6 (2–7)
Poor uniformity of body weights of the pullet flock	5.6 ± 0.18	6 (3–7)
Diseases	5.5 ± 0.25	6 (2–7)
Ectoparasites	5.4 ± 0.25	6 (2–7)
Light evenness	5.3 ± 0.24	6 (2–7)
Bird density	5.3 ± 0.27	5 (2–7)
Mortality	5.3 ± 0.23	6 (2–7)
Endoparasites	5.2 ± 0.26	6 (2–7)
Feed quality	5.1 ± 0.26	6 (1–7)
Inappropriate choice of lamps	5.1 ± 0.33	6 (1–7)
Hot summer weather	5.1 ± 0.23	6 (1–7)
Origin of the birds	5.1 ± 0.27	5 (1–7)
Natural light	5.1 ± 0.26	5 (1–7)
Production system	4.9 ± 0.26	5 (1–7)
Time spent on observing flock	4.9 ± 0.22	4 (2–7)
Feed changes	4.9 ± 0.24	5 (1–7)
Air ammonia	4.6 ± 0.22	5 (1–7)
Temperature	4.5 ± 0.18	5 (2–6)
Location of windows	4.5 ± 0.33	5 (1–7)
Floury feed	4.5 ± 0.21	5 (1–7)
Power failure	4.5 ± 0.36	5 (1–7)
Air humidity	4.4 ± 0.24	5 (1–7)
Change of manager	4.3 ± 0.24	4 (1–7)
Transportation age	4.2 ± 0.27	4 (1–7)
Breed	4.2 ± 0.24	4 (1–7)
Vaccinations	4.2 ± 0.31	4 (1–7)
Air dustiness	4.1 ± 0.40	4 (1–7)
Litter condition	4.1 ± 0.26	4 (1–7)
Medications	4.1 ± 0.28	4 (1–7)
Temporary care taker	4.1 ± 0.23	4 (1–7)
Perches (location, functionality)	3.9 ± 0.28	4 (1–7)
Season	3.7 ± 0.21	4 (1–6)
Thunder	3.7 ± 0.33	4 (1–7)
Nests (location, functionality)	3.6 ± 0.23	4 (1–6)
Litter quantity	3.5 ± 0.25	4 (1–6)
Litter material	3.4 ± 0.25	4 (1–6)
Litter addition	3.4 ± 0.22	4 (1–6)

* Scale from 1 (not important) to 7 (extremely important).

**Table 4:** The most important methods to prevent pecking problems and intervention measures to manage an ongoing pecking problem, according to Finnish egg producers.

Measure *	Prevention		Intervention	
	Number of First Places	Total Points	Number of First Places	Total Points
Optimal feedeing	12	116	9	94
Optimal lighting	12	111	15	135
Housing conditions **	2	102	1	46
Avoiding natural light leakage	2	14	1	13
Flock management ***	1	23	2	26
Removal of the pecker	0	0	2	27
Removal of the victim	0	0	2	25
Successful rearing period	1	19	0	0
Breed	1	11	0	0
Flock size	1	5	0	0
Additional foraging material	0	0	1	33
Additional salt (NaCl)	0	0	1	10

* The respondents listed the five most important methods, in order of importance, to prevent pecking problems, and intervention measures. To rate all mentioned factors, the most important factor received five points, and the least important factor one point. For analyses, all points were summed to get total points given to each factor. ** Housing conditions included ventilation, air quality, minimal dustiness, optimal humidity and temperature. *** Flock management included flock observation, management and management skills.

**Table 5:** The importance of preventive and intervention measures for pecking-related problems in laying hens, estimated by Finnish egg producers.

Measure	Mean *	Median (Range) *
Removal of the pecker	5.6 ± 0.30	6 (1–7)
Removal of the victim	4.3 ± 0.28	4 (1–7)
Straw bales	4.1 ± 0.94	3 (1–7)
Hanging string	3.7 ± 0,98	3 (2–7)
Pecking stones	3.4 ± 0.81	3 (1–7)
Hanging objects	3.1 ± 0.74	3 (1–7)

* Scale from 1 (not important) to 7 (extremely important).

## Supplementary Materials

The following are available online at http://www.mdpi.com/2076-2615/9/2/43/s1, **Table S1**. Questions to Finnish egg producers about pecking-related problems in laying hen flocks.
